# Clinical Lecture in the U. S. Marine Hospital

**Published:** 1852-11

**Authors:** W. B. Herrick


*Clinical Lecture, in the U. S. Marine Hospital, by* W. B. HERRICK, M. D.

THE patient before us has been sick nine weeks—was treated at Cleveland, probably for pneumonia—he partly recovered, but continued to cough, raising blood at times—has had pain in the chest and right side, has had little or no appetite, occasional chills followed by fever, and has felt himself constantly losing strength.

You will observe that his skin is pale, tongue slightly furred—he is emaciated, and presents the appearance of great debility.

On an examination of his chest, we find the upper portions of both lungs resonant on percussion, the lower lobes somewhat dull, especially on the right side. The liver extends below the ribs, where its free edge is distinctly felt, across into the epigastric and left hypochondriac regions. It is tender on pressure.

Auscultation reveals the natural respiratory murmur in the upper portions of the lungs, deficient or obscured respiratory sounds in the lower portions, and the mucous ronchus at the line of separation between the resonant and dull portions.

There has evidently been inflammation of the parenchyma of portions of both lungs, complicated with hepatitis. At the present stage of the affection, it is difficult to say whether the liver or lungs became first implicated, or whether both were simultaneously affected.

A few days previous to his admission, he had a new attack of pulmonary inflammation. The pulse was quick and full, tongue coated, skin hot and dry, and urinary secretion scanty. Auscultation showed the usual sounds of pneumonia. The matter expectorated was tinged with blood. He was ordered chloride of sodium ʒj, opii. grs. iij, every four hours. In 24 hours the inflammatory symptoms had nearly all subsided, and yesterday he presented very much the appearance you see this morning.

We will take this occasion to make a few remarks on what we believe to be the pathology and indications of treatment in pneumonia. In order that we may have clear ideas of the changes which take place, we will call your attention to the physiological relations of assimilation, and excretion. For the sake of distinctness, we shall use the term primary assimilation to indicate all the changes that take place in food in the alimentary canal, including chymification and chylification, or until it is converted into perfectly elaborated blood. By secondary assimilation, we shall understand the chemico-vital changes by which the nutritious material in the blood-vessels is taken up and deposited, to form the nervous, muscular, fibrous, cartilaginous, and osseous structures. We have here two stages of assimilation, or the introduction and appropriation of food; but this food having subserved the purpose of the economy, must be removed, and the process by which it is thrown off may also be divided into two stages, which, for convenience, we shall call primary and secondary excretion, understanding by the first, the removal of the effete particle, and its transfer into the blood-vessels; and by the second, its elimination from the circulating fluids, by the excreting organs, such as the lungs, liver, kidneys, skin, &c.

When the building up and breaking down processes are equal, or when excretion and assimilation sustain to each other their proper relation, the system is in a condition of health. Any disturbance of this equilibrium, with the exception of that of growth, constitutes disease.

In pneumonia, and most acute diseases, we believe primary excretion is more active than secondary assimilation, by which there comes to be a wasting of the tissues, and an accumulation in the blood-vessels, of fibrine and excretable matters. And as oxygen is the agent by which the breaking down process is effected, it is evident that we may affect the disease by modifying the amount of oxygen taken into the system. This may be accomplished by abstracting from the system, by venesection, the blood globules, the oxygen carries, but in doing so we also abstract a portion of the nutritious material for the supply of the tissues. It is evident that we may also lessen the amount of oxygen introduced, by reducing the frequency of respiration, and the rapidity of the circulation.

In the treatment of a case of acute inflammation, we have, therefore, to consider the peculiar condition of the system, as pointing to one or all of these means, for the purpose of fulfilling the indications. In patients of strong, healthy constitution, in which there is an abundant supply of blood, rich in albumen, we may resort to blood-letting, followed by other agents calculated to reduce the frequency of respiration and pulsation. But in patients already enfeebled by disease, in whom the blood is impoverished, and the assimilating forces weakened, it seems to us hazardous to reduce the quality of the blood still more, and weaken to a greater extent, the life forces. We prefer, therefore, in such cases, to endeavor to meet the indications, by acting on respiration and circulation in some other manner than by direct depletion, and for this purpose, opium is perhaps better adapted than any other agent with which we are acquainted.

Given in full doses, by which we mean from three to six grains, repeated every four to eight hours, it not only modifies the respiratory and circulatory movements, but also acts upon the excreting organs, as the skin, kidneys, &c. This is especially the case if combined with full doses of quinine. In this manner we believe we are able to maintain, as nearly as may be, the healthy constitution of the blood itself, while we correct the exaggerated primary excretion, without impairing secondary assimilation.

There may be normal assimilation, and exaggerated excretion; there may also be normal excretion, and deficient assimilation, constituting a lower grade of inflammation.

This latter condition is frequently met with in patients who have been exposed to miasmatic influences, whatever they may be.

One prominent feature in a majority of diseases that we are called to treat, is anæmia, impoverished blood, and torpidity of the organs concerned in secondary excretion. This is especially the case in the patient before us. The skin was dry and rough, the liver congested and inflamed, and therefore incapable of performing its function ; the secretion of the kidneys scanty, and the lungs throughout nearly one half of their extent, so much diseased as to be unable to eliminate the usual amount of carbon. The inflammatory symptoms were subdued by opium. The skin was excited by opium, combined with quinine. The liver and kidneys have been stimulated by the alkali soda, in combination with chlorine, which, as well as iodine, seems to us to be capable of taking the place of oxygen in the blood.

The future treatment must consist of tonics, and a supporting diet. Iron, to increase the blood globules, food, rich in albumen, to nourish the tissues, and alkalies, to keep up the action of the secreting organs, and especially to keep the biliary secretion in a soluble condition, and thus favor the reduction of the hepatic congestion.

We have another case here, affected with pleuritis, with a slight pneumonic complication. This patient had been under treatment several days, for intermittent fever; the paroxysms had been arrested, but he was still very much debilitated. Two days since, he was attacked with pleuritis. At the time of the morning visit, his pulse was 120 per minute, full and strong; the skin hot and dry ; the tongue coated; constant disposition to cough, but extreme pain on making the least effort to do so ; respiration abdominal; face flushed; countenance indicating much anxiety and distress. Percussion showed slight dulness over the region of the left lung.—On listening for the respiratory murmur, we found it obscured by a very distinct friction sound, revealing pleuritic inflammation, which already seemed to have invaded the whole of the left side. The inflammation was active, but a few hours had elapsed since its first development. The pain and anxiety were intense. The tendency here was evidently to exaggerated primary excretion, the particle was seized upon as if by force, and thrown from its position, while its place was not supplied by new material. There had also been, for some time, deficient action of the secondary excreting organs, the blood was loaded with effete matters, while at the same time, it was wanting in albumen, and perhaps other nutritious materials.

We had here two ends to be accomplished. First, to arrest the excessive action of the breaking down force; and secondly, to aid the organs concerned in secondary excretion, in their elimination from the system, of the worn out matter.

To meet the first indication, we prescribed opium, grs. jv., every six hours. To meet the second, we combined with each dose, quinine, grs. vij., ipecac, grs. j., with a view to its acting on the skin, and probably other excreting organs.

Yesterday morning, after being under that treatment twenty-four hours, we found him with a pulse 100 per minute, soft and compressible, the skin moist, and possessed of that peculiar, clammy feel, so frequently noticed in profuse perspiration from quinine and ipicac; the pain in the chest almost entirely relieved; a slight rubbing sound was heard at one point only, breathing free and easy, tongue coated, dark and dry along the centre, still some cough, sputa slightly tinged with blood.

R. Sulph. Quinine grs. xxjv.
Opii. grs. xvj.
Chlo. Sodii. ʒjv.
M. ft. Chart. No. jv.
Take one every 6 hours.

This morning, as you will perceive, the skin is slightly moist, but cool; the tongue is clean and moist, looks as in health, complains of no pain in the chest, a slight roughness of the respiratory murmur is the only abnormal sound that we are able to detect, the pulse is not more than 80 per minute, rather small and feeble, the secretions of the kidneys and liver seem to be normal. In a word, convalescence seems to be fully established. We shall prescribe for him iron and good nutritious diet, with dilute nitro mur. acid, to assist in the digestion of food.

